# Toxic Effects of Povidone-Iodine on *Macrobrachium rosenbergii*: Concentration-Dependent Responses in Oxidative Stress, Immunosuppression, and Recovery Potential

**DOI:** 10.3390/ani15152196

**Published:** 2025-07-25

**Authors:** Tianhui Jiao, Yakun Wang, Jie Wei, Sikai Xu, Qiaoyan Zhou, Xidong Mu, Lingyun Yu

**Affiliations:** 1 Key Laboratory of Tropical and Subtropical Fishery Resources Application and Cultivation, Ministry of Agriculture and Rural Affairs, Pearl River Fisheries Research Institute, Chinese Academy of Fishery Sciences, Guangzhou 510380, China; jiaotianhui0730@163.com (T.J.); wykzkyky@163.com (Y.W.); weijie929ezio@outlook.com (J.W.); 2023710942@yangtzeu.edu.cn (S.X.); zhouqiaoyan@prfri.ac.cn (Q.Z.); muxd@prfri.ac.cn (X.M.); 2 College of Fisheries and Life Science, Shanghai Ocean University, Shanghai 201306, China

**Keywords:** disinfectant, povidone-iodine, aquaculture industry, *Macrobrachium rosenbergii*, gills, mitochondrial apoptosis pathway, immunosuppression, restorative capacity, oxidative stress

## Abstract

Povidone-iodine (PVP-I), a widely used aquaculture disinfectant, remains poorly understood in terms of sublethal toxicity and damage reversibility. This study employed *Macrobrachium rosenbergii* as the model organism to evaluate the acute toxicity and sublethal effects of PVP-I through a 4-day exposure experiment followed by a 7-day depuration period. Acute toxicity tests enabled the determination of 24–96 h median lethal concentrations (LC_50_), with the 96 h LC_50_ being 5.67 mg/L and the safe concentration (SC) being 1.37 mg/L. Based on this, three sublethal concentrations (1.14, 1.89, and 2.84 mg/L) were tested over a 4-day exposure followed by a 7-day depuration period. Investigated endpoints included gill ultrastructure, apoptosis, and antioxidant and immune-related gene expression. Subacute exposure at 1.89 and 2.84 mg/L induced mitochondrial vacuolization, upregulated apoptosis-related genes (*Cyt-c*, *Caspase-3*, *Bok*), and downregulated antioxidant gene expression (*SOD*, *CAT*, *GSH-Px*). The high-concentration group also showed sustained Toll-like receptor (*Toll*) gene overexpression and acid phosphatase (*ACP*) gene suppression. After depuration, antioxidant gene expression normalized; however, apoptotic markers in gill tissue remained impaired. Overall, high PVP-I concentrations cause irreversible gill damage via mitochondrial-mediated apoptosis, whereas lower concentrations (≤1.14 mg/L) allow for greater recovery. These results offer crucial toxicodynamic insights for safer PVP-I use and risk assessment in *M. rosenbergii* aquaculture.

## Simple Summary

Povidone-iodine (PVP-I) is a widely used disinfectant in *Macrobrachium rosenbergii* aquaculture. This study, by analyzing changes in gill tissue ultrastructure, cellular apoptosis, and antioxidant and immune-related genes in the hepatopancreas under 4-day PVP-I exposure and 7-day recovery in clean water conditions, investigates the sublethal toxicity mechanisms and damage reversibility of PVP-I on *M. rosenbergii* in order to provide a basis for standardized farming and safe drug use in *M. rosenbergii* and to provide references for toxicity evaluation of PVP-I on crustaceans.

## 1. Introduction

In recent years, global aquaculture has expanded rapidly, becoming a critical source of animal protein [1]. However, intensified farming practices have led to increased water eutrophication and the proliferation of pathogenic microorganisms, resulting in frequent disease outbreaks in farmed animals that hinder sustainable development [2,3]. Historically, antibiotics were widely used in aquaculture to manage disease [4]; due to weak intestinal absorption and incomplete metabolism in animals, most antibiotics are excreted as unchanged active compounds and metabolites through feces and urine [5]. Consequently, their excessive usage has caused persistent residues in aquatic environments, contributing to antimicrobial resistance in farmed species [6] and posing significant human health risks via bioaccumulation in food chains [7]. These concerns have prompted a shift toward non-antibiotic disease management strategies [8]. Disinfectants with broad-spectrum antibacterial activity and lower environmental persistence are now integral to disease management protocols.

Disinfectants commonly used in aquaculture include formaldehyde [9], peracetic acid [10], chlorine-based bleach [11], and PVP-I [12] (Table S1). Among these, the antimicrobial efficacy of PVP-I stems from the release of free iodine, which disrupts microbial metabolic pathways [13]. It is effective against bacteria [11,14,15], fungi [16], parasites [17], and certain spore forms [18]. However, the ecotoxicological risks of PVP-I to farmed aquatic species warrant further attention. Studies show that its toxicity is both concentration- and time-dependent. For instance, short-term low-concentration exposure (e.g., 3 days at 4.38 mg/L) enhanced immune function and antioxidant capacity in *Eriocheir sinensis*, conferring improved resistance against *Aeromonas hydrophila* [12]. Conversely, chronic low-dose exposure (e.g., 30 days at 50 µg/L) induces oxidative stress through disinfectant deposition in the liver of *Ctenopharyngodon idellus* and modulates inflammatory responses by suppressing mRNA expression levels of immune factors in hepatic tissues [19]. Meanwhile, short-term high-intensity exposure (e.g., 7.5 mL/m^3^ for 30 min) disrupts the microbial architecture of branchial biofilms in *C. carpio*, suppresses lysozyme activity, and leads to incomplete recovery of immune function even after a 7-day depuration period [20]. The excessive use of disinfectants not only causes damage to cultured organisms but also poses risks to future consumers (Table S2). Therefore, a comprehensive understanding of its toxicological mechanisms and recovery potential is essential for the responsible and standardized use of PVP-I in aquaculture.

*Macrobrachium rosenbergii*, native to tropical and subtropical regions, is the world’s most widely farmed freshwater prawn species [21]. In recent years, the spread of various pathogens has increasingly threatened its sustainable cultivation [22]. Although PVP-I has been utilized for disease control in *M. rosenbergii* aquaculture, its safety concentration threshold, sublethal toxic effects, and damage reversibility remain undefined. To address this knowledge gap, this study will investigate the impact and reversibility of PVP-I exposure on the antioxidant system, immune competence, gill tissue ultrastructure, and apoptosis in *M. rosenbergii*, thereby providing scientific foundations for standardized farming practices and safe chemical application in giant freshwater prawn cultivation.

## 2. Materials and Methods

### 2.1. Experimental Materials and Maintenance

Juvenile *M. rosenbergii* were obtained from the Pearl River Fisheries Research Institute, Chinese Academy of Fishery Sciences. Individuals were selected based on good clinical health and vigor (mean body weight: 1.44 ± 0.13 g). Prior to experimentation, prawns were acclimated for 7 days in concrete tanks under conditions simulating the experimental environment. During acclimation, a restricted basal diet was provided to facilitate physiological adjustment and reduce potential confounding from environmental fluctuations. A PVP-I solution containing 11% available iodine (CAS No. 25655-41-8; Shanghai Macklin Biochemical Technology Co., Ltd., Shanghai, China) was freshly prepared immediately before use. All experimental protocols were reviewed and approved by the Animal Bioethics Committee, Pearl River Fisheries Research Institute, Chinese Academy of Fishery Sciences, China (Approval No. LAEC-PRFRI-2024-05-03, Approval Date: 3 May 2024).

### 2.2. Median Lethal Concentration (LC_50_) of PVP-I on M. rosenbergii

Acute toxicity testing involved seven PVP-I concentrations (0, 4.5, 5.0, 5.5, 6.0, 6.5, and 7.0 mg/L), with three replicates per treatment. Each replicate consisted of 30 prawns maintained in 50 L aquaria under controlled conditions for a 96 h exposure period. To ensure consistent water quality, 50% of the tank water volume was replaced daily with freshly prepared PVP-I solutions of the corresponding concentration. To minimize handling stress, replacement water was equilibrated for 10 min before being added. Survival rates were recorded at 24, 48, 72, and 96 h.

### 2.3. PVP-I Exposure Experiment

Based on the 96 h median lethal concentration (LC_50_ = 5.67 mg/L) determined from the acute toxicity test, three sublethal concentrations, 1.14 mg/L (1/5 LC_50_), 1.89 mg/L (1/3 LC_50_), and 2.84 mg/L (1/2 LC_50_), were selected for subacute exposure. A clean water control group (0 mg/L) was also included. Each treatment group contained 90 prawns (three replicates of 30). After a 4-day exposure period, all groups were transferred to clean water for a 7-day depuration recovery period. Water replacement followed the same procedure used during acute toxicity testing. Sampling was performed on day 4 (immediately after exposure) and day 11 (post-depuration). At each time point, 27 prawns per group (9 per replicate) were randomly selected for analysis. Gill tissues were divided into three parts: one fixed in 2.5% glutaraldehyde for transmission electron microscopy (TEM), another fixed in 4% paraformaldehyde for 24 h for paraffin sectioning, and the remaining portion flash-frozen in liquid nitrogen for RNA extraction and enzymatic assays. Hepatopancreas tissues were also flash-frozen and stored at −80 °C for subsequent RNA extraction and enzymatic assays.

### 2.4. RNA Extraction, cDNA Synthesis, and Real-Time Quantitative PCR (qPCR)

Total RNA was extracted using the Eastep Super Total RNA Extraction Kit (Promega, Beijing, China), following the manufacturer’s protocol. RNA concentration and purity were assessed spectrophotometrically (OD_260_/OD_280_) using a Nanodrop 8000 spectrophotometer (Thermo Fisher Scientific, Waltham, MA, USA). RNA integrity was confirmed via 1.0% agarose gel electrophoresis. cDNA was synthesized from total RNA using the M-MLV Reverse Transcriptase Kit (Invitrogen, Waltham, MA, USA) according to the manufacturer’s instructions. Both RNA and cDNA samples were stored at −80 °C in an ultra-low-temperature freezer until further analysis. Primers for target and reference genes were designed based on conserved sequences from *M. rosenbergii*. Their specificity and amplification efficiency were validated prior to use (primer sequences are provided in Table 1). Quantitative real-time PCR (qPCR) was performed using the StepOnePlus Real-Time PCR System (Applied Biosystems, Foster City, CA, USA). Each 20 μL reaction contained 10 μL iTaq Universal SYBR Green Supermix, 1 μL each of forward and reverse primers, 1 μL cDNA (5 ng/μL), and 7 μL ddH_2_O. The thermal cycling protocol was as follows: initial denaturation at 95 °C for 3 min; 35 cycles of 95 °C for 40 s (denaturation), 60 °C for 45 s (annealing), and 72 °C for 30 s (extension); and a final extension at 72 °C for 10 min. Relative gene expression was calculated using the 2^−ΔΔCt^ method [23], with each group analyzed in triplicate.

### 2.5. Determination of Antioxidative Parameters

Hepatopancreas tissue samples were homogenized in ice-cold physiological saline (0.86% NaCl) at a 1:9 (*w*/*v*) ratio using a homogenizer under ice-bath conditions. The homogenate was centrifuged at 12,000× *g* for 15 min at 4 °C. The resulting supernatant was aliquoted and stored at −80 °C for enzymatic analysis. Superoxide dismutase (SOD) activity was measured using the xanthine oxidase method. Catalase (CAT) activity was assessed using the ammonium molybdate method, whereas glutathione peroxidase (GSH-Px) activity was determined using a colorimetric assay. All reagents were obtained from commercial kits provided by Nanjing Jiancheng Bioengineering Institute (Nanjing, China). Sample preparation, reagent handling, and enzymatic analyses strictly followed the manufacturer’s instructions to ensure accuracy and reproducibility.

### 2.6. TUNEL Assay

Separate sections of their gills were fixed with 4% paraformaldehyde for 24 h and placed in ethanol at varying concentrations for dehydration. The tissue samples were prepared and set in paraffin wax after collection [25]. Cellular apoptosis was evaluated using the terminal deoxynucleotidyl transferase-mediated dUTP-biotin nick end labeling (TUNEL) assay, in accordance with the protocol of the apoptosis detection kit (Wuhan Servicebio Technology Co., Ltd., Wuhan, China) [26]. Paraffin-embedded tissue sections were deparaffinized, rehydrated, and subjected to antigen retrieval using proteinase K. Sections were then incubated with equilibration buffer at 25 °C for 10 min, followed by application of a reaction mixture containing TdT enzyme, fluorescein-dUTP, and reaction buffer (mixed at a 2:5:50 ratio) at 37 °C for 1 h. Nuclei were counterstained with DAPI (4′,6-diamidino-2-phenylindole) in the dark at 25 °C for 10 min. Slides were mounted using antifade medium and imaged using a fluorescence microscope. Viable nuclei exhibited blue fluorescence under UV excitation, whereas apoptotic nuclei (TUNEL-positive) appeared red.

### 2.7. Transmission Electron Microscopy (TEM)

Gill tissues were sectioned into small blocks (approximately 1 mm^3^) and fixed in 2.5% glutaraldehyde at 4 °C for 4 h. Samples were then rinsed thoroughly in 0.1 mol/L phosphate buffer to remove residual fixative. Tissue dehydration was performed using a graded ethanol series (50%, 70%, 80%, 90%, 95%, and 100%), with 20 min incubations at each step. Subsequently, propylene oxide was used for solvent exchange prior to infiltration with epoxy resin. The tissues were embedded in epoxy resin within embedding molds and polymerized at 25 °C. Ultrathin sections (approximately 70 nm) were obtained using an ultramicrotome, stained with lead citrate (5 min) and uranyl acetate (15 min), and examined using a transmission electron microscope (HT7800, Hitachi, Tokyo, Japan) to assess mitochondrial ultrastructure [27].

### 2.8. Statistical Analysis

Probit regression analysis [28] was conducted using SPSS 24.0 software (SPSS Inc., Armonk, NY, USA) to calculate the median lethal concentrations (LC_50_) of PVP-I at 24, 48, 72, and 96 h, along with the corresponding 95% confidence intervals. The safe concentration (SC) was calculated using the Turubell formula [29]: SC = (48 h LC_50_ × 0.3)/(24 h LC_50_/48 h LC_50_)^2^. All statistical analyses were conducted using GraphPad Prism 9 software (GraphPad Software, San Diego, CA, USA). Group differences were assessed using one-way analysis of variance, and results were reported as mean ± standard error (SE). Statistical significance was determined as follows: *p* > 0.05 (not significant), *p* < 0.05 (significant), and *p* < 0.01 (highly significant).

## 3. Results

### 3.1. Acute Toxic Effects of PVP-I on M. rosenbergii

Acute toxicity tests revealed that the 24 h, 48 h, 72 h, and 96 h LC_50_ values for PVP-I exposure in *M. rosenbergii* were 8.49, 6.90, 6.08, and 5.67 mg/L, respectively (Table 2). The calculated SC was 1.37 mg/L. During high-concentration exposure (≥6 mg/L), *M*. *rosenbergii* exhibited a distinct stress response: during the early stages of the experiment, the shrimp showed a significant increase in swimming speed, whereas in the later stages, their activity sharply declined, followed by a prostrate state on the bottom of the tank. Mortality increased in a concentration- and time-dependent manner, with statistically significant differences observed across exposure levels (*p* < 0.01). While no mortality occurred in the control group (0 mg/L), all treatment groups showed a significant positive correlation among mortality, PVP-I concentration, and exposure duration.

### 3.2. Expression of Hepatopancreatic Immunity-Related Genes

**Figure 1:**
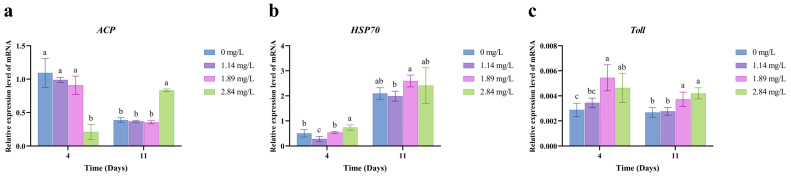
Effect of PVP-I with different concentrations on the relative mRNA expression of immune-related genes in hepatopancreas tissues of *M. rosenbergii*. (**a**) Relative mRNA gene expression of *ACP*; (**b**) Relative mRNA gene expression of *HSP70*; (**c**) Relative mRNA gene expression of *Toll*. Different letters indicate significant differences among experimental groups at the same time point (*p* < 0.05). Data are presented as the mean ± standard error (SE) (*n* = 3).

After 4 days of PVP-I exposure, *M. rosenbergii* exhibited concentration-dependent changes in the expression of immune-related genes in the hepatopancreas (Figure 1). In the high-concentration group (2.84 mg/L), acid phosphatase (*ACP*) expression was significantly lower than in the control and other treatment groups (*p* < 0.05), suggesting that high-concentration PVP-I exposure may cause severe damage to the immune system of *M*. *rosenbergii*. However, following 7 days of depuration, *ACP* expression in this group rebounded and exceeded the levels in all other groups (*p* < 0.05). While the heat shock protein 70 gene (*HSP70*) expression was the lowest in the low-concentration group (1.14 mg/L) (*p* < 0.05), its expression was significantly upregulated in the high-concentration group (2.84 mg/L) during exposure (*p* < 0.05). After recovery, *HSP70* expression returned to levels comparable to those in the control group (*p* > 0.05). Toll-like receptor (*Toll*) gene expression was significantly higher in the medium (1.89 mg/L) and high-concentration (2.84 mg/L) groups than in the control group (*p* < 0.05), and this upregulation persisted after depuration (*p* < 0.05).

### 3.3. Antioxidant System Response in the Hepatopancreas

**Figure 2:**
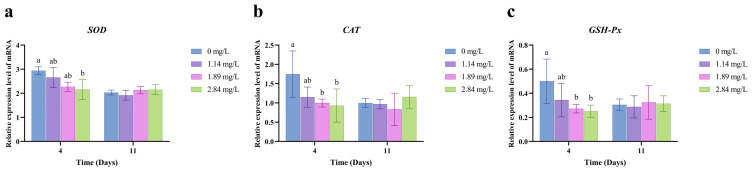
Effect of PVP-I with different concentrations on the relative mRNA expression of antioxidant-related genes in hepatopancreas tissues of *M. rosenbergii*. (**a**) Relative mRNA gene expression of *SOD*; (**b**) Relative mRNA gene expression of *CAT*; (**c**) Relative mRNA gene expression of *GSH-Px*. Different letters indicate significant differences among experimental groups at the same time point (*p* < 0.05). Data are presented as the mean ± standard error (SE) (*n* = 3).

PVP-I exposure led to concentration-dependent changes in the expression of antioxidant-related genes in the hepatopancreatic tissue (Figure 2). After 4 days, the high-concentration group (2.84 mg/L) exhibited significant downregulation of *SOD*, *CAT*, and *GSH-Px* genes (*p* < 0.05). In the medium-concentration group (1.89 mg/L), *CAT* and *GSH-Px* expression was also significantly reduced (*p* < 0.05). Notably, after 7 days of depuration, gene expression levels in all groups returned to values statistically indistinguishable from those of the control group (*p* > 0.05).

**Figure 3:**
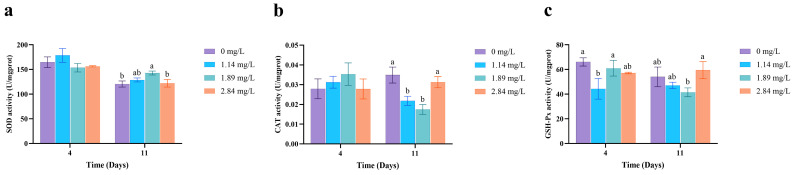
Effect of PVP-I with different concentrations on the antioxidant enzymes activities in the hepatopancreas tissues of *M. rosenbergii*. (**a**) SOD activity; (**b**) CAT activity; (**c**) GSH-Px activity. Different letters indicate significant differences among experimental groups at the same time point (*p* < 0.05). Data are presented as the mean ± standard error (SE) (*n* = 3).

Enzymatic activity assays (Figure 3) showed that in the low-concentration group (1.14 mg/L), GSH-Px activity was significantly reduced (*p* < 0.05) and did not fully recover after 7 days (*p* < 0.05). In the high-concentration group (2.84 mg/L), CAT activity remained significantly lower than in the control group post-recovery (*p* < 0.05). Conversely, SOD activity in the medium-concentration group (1.89 mg/L) after recovery was significantly higher than that in the control group (*p* < 0.05).

### 3.4. Expression of Apoptosis-Related Genes in Gill Tissues

**Figure 4:**
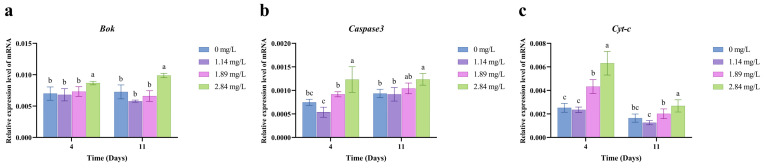
Effect of PVP-I with different concentrations on the relative mRNA expression of apoptosis-related genes in gill tissues of *M. rosenbergii*. (**a**) Relative mRNA gene expression of *Bok*; (**b**) Relative mRNA gene expression of *Caspase3*; (**c**) Relative mRNA gene expression of *Cyt-c*. Different letters indicate significant differences among experimental groups at the same time point (*p* < 0.05). Data are presented as the mean ± standard error (SE) (*n* = 3).

**Figure 5:**
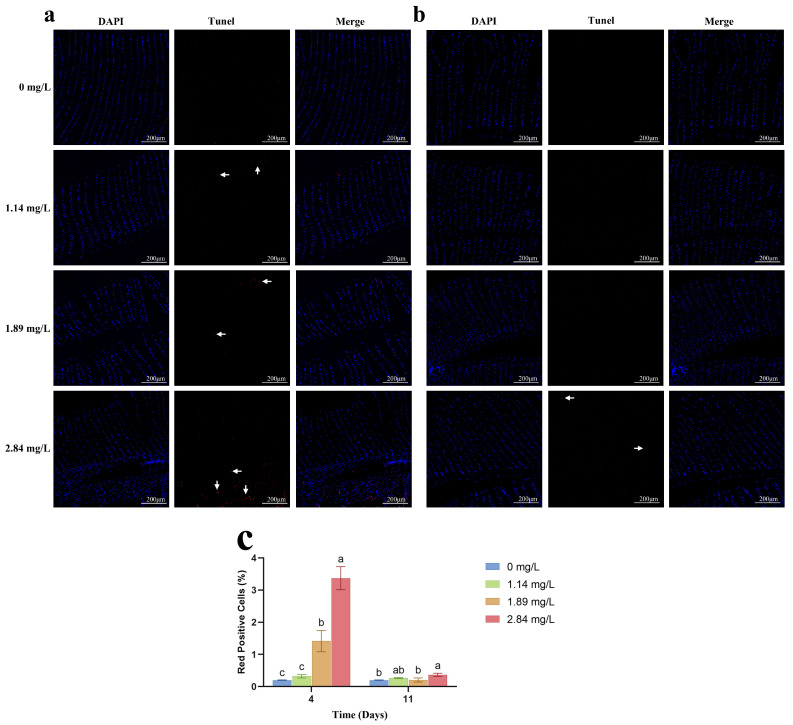
Apoptosis detection in the gills of *M. rosenbergii* exposed to varying PVP-I concentrations. (**a**) TUNEL staining showing apoptosis in red after exposure to different PVP-I concentrations after 4 days of clear water farming. (**b**) TUNEL staining showing apoptosis in red after exposure to different PVP-I concentrations after 7 days of clear water farming. (**c**) Red fluorescence positivity rate of TUNEL signals in different groups. Live gill cell nuclei show blue signals. TUNEL-positive gill cell nuclei display red signals (arrow). Different letters indicate significant differences among experimental groups at the same time point (*p* < 0.05). Data are presented as the mean ± standard error (SE) (*n* = 3).

The effects of PVP-I exposure on apoptosis-related gene expression in the gills of *M. rosenbergii* are shown in Figure 4. After 4 days of exposure, the high-concentration group (2.84 mg/L) exhibited significant upregulation of B cell lymphoma 2 ovarian killer (*Bok*), *Caspase-3*, and cytochrome c (*Cyt-c*) (*p* < 0.05), suggesting that excessive concentrations of PVP-I can induce apoptosis in the gill tissues of *M. rosenbergii*. Notably, the expression of these genes remained significantly elevated compared to that in the control group even after the 7-day depuration period (*p* < 0.05). In the medium-concentration group (1.89 mg/L), *Cyt-c* expression was also significantly upregulated after 4 days (*p* < 0.05) but returned to control levels post-recovery (*p* > 0.05). Apoptotic activity in gill tissues, as assessed using TUNEL staining (Figure 5), revealed a significant increase in TUNEL-positive signals (red fluorescence) in the high-concentration group after 4 days of exposure (*p* < 0.05). Residual apoptotic signals persisted in this group even after recovery (Figure 5c), indicating incomplete reversal of apoptosis.

### 3.5. Ultrastructural Damage to Gill Tissue

**Figure 6:**
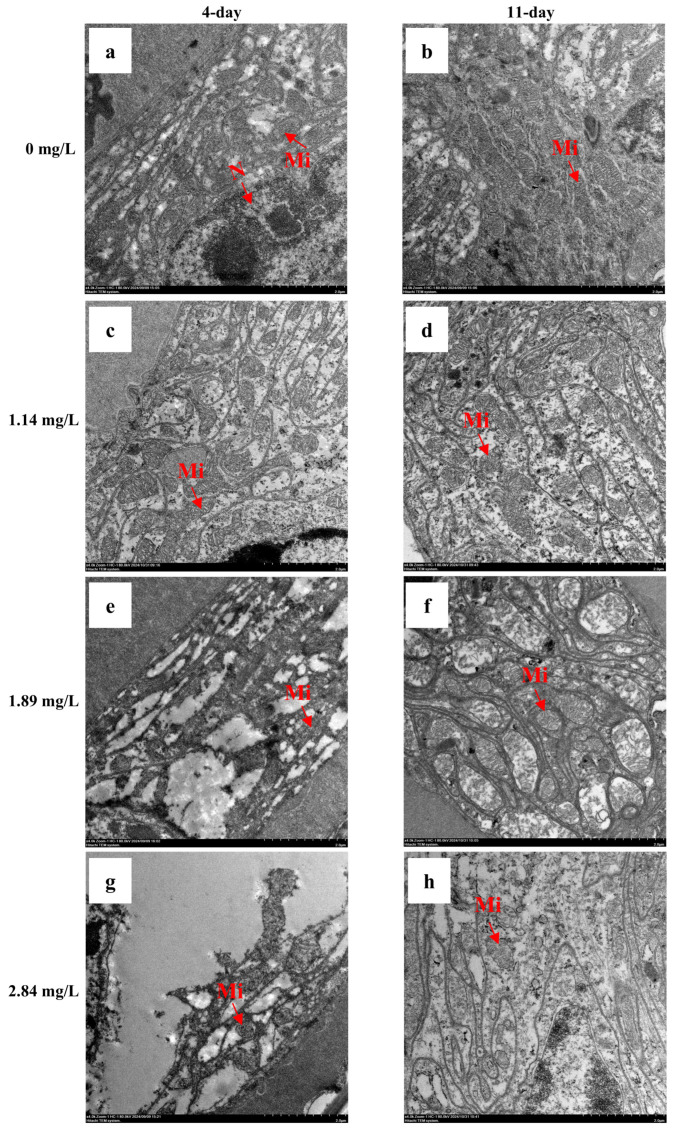
Ultrastructure of gill filament under transmission electron microscopy. (**a**,**b**) The 0 mg/L group. (**c**) The 1.14 mg/L group after 4 days of PVP-I exposure. (**e**) The 1.89 mg/L group after 4 days of PVP-I exposure. (**g**) The 2.84 mg/L group after 4 days of PVP-I exposure. (**d**) The 1.14 mg/L group after 7 days of clear water farming. (**f**) The 1.89 mg/L group after 7 days of clear water farming. (**h**) The 2.84 mg/L group after 7 days of clear water farming. Mt: mitochondrion; Mi: mitochondrion; and N: nucleus.

TEM revealed concentration-dependent ultrastructural changes in the gill mitochondria of *M. rosenbergii* (Figure 6). In the control group, mitochondria exhibited intact morphology with a regular oval shape and uniform matrix distribution (Figure 6a,b). After 4 days of exposure, the low-concentration group (1.14 mg/L) showed no apparent structural differences from the controls (Figure 6c). Conversely, the medium (1.89 mg/L) and high-concentration (2.84 mg/L) groups exhibited dose-dependent mitochondrial damage, including vacuolization and matrix disorganization, with severity increasing alongside PVP-I concentration (Figure 6e,g). Following 7-day depuration, partial restoration of mitochondrial integrity was observed in the low- and medium-concentration groups (Figure 6d, f). In contrast, the high-concentration group retained significant mitochondrial lesions (most notably, persistent vacuoles and cristae fragmentation), suggesting limited recovery from structural damage at higher exposure levels (Figure 6h).

## 4. Discussion

PVP-I, a coordination complex of polyvinylpyrrolidone and elemental iodine, exhibits broad-spectrum antimicrobial activity against bacteria, fungi, mold spores, and certain enveloped viruses [30]. In this study, the acute toxicity of PVP-I to *M. rosenbergii* was systematically evaluated. The 24 h, 48 h, 72 h, and 96 h LC_50_ were 8.49, 6.90, 6.08, and 5.67 mg/L, with a calculated SC of 1.37 mg/L. The SC (1.37 mg/L) for *M. rosenbergii* was significantly higher than the recommended therapeutic range (0.045–0.075 mg/L) specified in the China Fishery Pharmaceutical Usage Regulations (SC/T 1132-2016). This suggests a relatively wide safety margin for *M. rosenbergii* under standard treatment conditions. When compared with other aquatic species, the SC for *M. rosenbergii* aligns closely with values reported for *Litopenaeus vannamei* (SC = 1.2 mg/L) [31], though it is significantly lower than that reported for *GIFT Oreochromis niloticus* (SC = 2.19 mg/L) [30]. These interspecies differences in sensitivity are likely attributed to inherent physiological and metabolic differences. Crustaceans, in particular, rely on direct substance exchange via the gill epithelium and lack an adaptive immune system, characteristic of vertebrates [32], making them more vulnerable to waterborne toxicants. In contrast, teleost fish benefit from more advanced hepatic detoxification processes and complex immune responses [33].

Crustaceans rely solely on their innate immune system to defend against pathogens [32], with *ACP*, *HSP70*, and *Toll* genes playing pivotal roles in immune defense and pathogen recognition. *ACP*, a lysosomal marker enzyme, functions in acidic environments to modify the surface properties of foreign particles, thereby enhancing phagocytic uptake and degradation by immune cells [34]. In this study, *ACP* expression in the hepatopancreas after 4 days of exposure to a high concentration of PVP-I (2.84 mg/L) was significantly lower than in the control and low-concentration groups. However, after a 7-day depuration period, *ACP* expression rebounded sharply, exceeding levels in all other groups. This suggests a compensatory response, likely driven by metabolic regulation within the immune system to counteract prior immunosuppressive effects. Persistent upregulation of *Toll* gene expression was also observed in the medium- and high-concentration groups (1.89 and 2.84 mg/L, respectively), with levels remaining elevated post-recovery. This may reflect continued recognition of residual pathogen-associated molecular patterns [35]. Although prolonged activation of Toll signaling could enhance pathogen clearance, it may also divert energy resources and promote chronic inflammation [36]. The transient upregulation of *HSP70* in high-concentration groups during initial exposure and its normalization post-recovery suggest that *HSP70* plays a protective role against short-term oxidative stress by maintaining proteostasis [37]. Collectively, the expression dynamics of *ACP*, *Toll*, and *HSP70* highlight the adaptive plasticity of the crustacean immune system. However, the persistent activation of *Toll* and the transient stress response of *HSP70* indicate that high concentrations of PVP-I impose dose-dependent and temporally cumulative effects on immune functionality.

PVP-I exposure also revealed a clear disconnect between the recovery of gene expression and enzymatic activity within the hepatopancreatic antioxidant system of *M. rosenbergii* (Figure 2 and Figure 3). In this study, after 4 days of exposure, the high-concentration group (2.84 mg/L) exhibited significant downregulation of *SOD*, *CAT*, and *GSH-Px* gene expression, whereas *CAT* and *GSH-Px* expression was also suppressed in the medium-concentration group (1.89 mg/L). These findings are consistent with findings in *Ctenopharyngodon idella* exposed to chronic PVP-I, which showed oxidative stress and secondary hepatotoxicity [19], likely due to oxidative interference with transcriptional regulation [38]. Following the 7-day depuration period, antioxidant gene expression levels in all groups returned to those of the control, suggesting high reversibility of transcriptional impairments. This recovery may be attributed to the restoration of redox homeostasis and reactivation of redox-sensitive transcription factors, such as nuclear factor erythroid 2-related factor 2 (Nrf2) [38]. These findings suggest that crustaceans can rapidly repair oxidative damage at the transcriptional level following short-term toxicant exposure. However, the recovery of enzymatic activity showed marked hysteresis and concentration-dependent delays. For example, GSH-Px activity in the low-concentration group (1.14 mg/L) failed to fully recover, CAT activity in the medium- and high-concentration groups remained below control levels, and SOD activity in the medium-concentration group was paradoxically elevated above baseline. Although the restoration of gene expression suggests that *M. rosenbergii* exhibits short-term stress resilience, the incomplete enzymatic recovery points to potential lingering oxidative damage. Therefore, in aquaculture practice, relying solely on transcriptional markers may lead to an underestimation of chronic toxicity risks. A more accurate assessment of sublethal stress should also integrate enzymatic activity data.

Prolonged oxidative stress triggers apoptosis through activation of intrinsic apoptotic pathways [39]. Here, high-concentration PVP-I exposure (2.84 mg/L) significantly induced apoptosis in the gill tissues of *M. rosenbergii*, as evidenced by the upregulation of key pro-apoptotic genes: *Bok*, *Caspase-3*, and *Cyt-c* (Figure 4). *Bok* promotes apoptosis by competitively binding anti-apoptotic proteins [40], while *Caspase-3*, the primary executioner caspase, is activated via *Cyt-c*-mediated apoptosome formation, marking the terminal phase of programmed cell death [41]. After 4 days of exposure, these genes were significantly upregulated in the high-concentration group, with *Cyt-c* also being elevated in the medium-concentration group (1.89 mg/L). These results are consistent with previous reports of PVP-I-induced apoptosis in hepatic and intestinal tissues of *Ctenopharyngodon idella* [19,25], suggesting that the oxidative properties of iodine drive apoptotic gene activation. Notably, even after a 7-day depuration period, the expression of apoptosis-related genes in the high-concentration group remained significantly higher than that in the control group, indicating persistent apoptotic signaling and likely irreversible damage. This conclusion was further corroborated by TUNEL staining, which revealed sustained elevated levels of apoptotic (red fluorescence-positive) cells in the high-concentration recovery group compared to those in the control group (Figure 5c).

The ultrastructure of gill tissues is widely recognized as a sensitive biomarker for assessing health status in aquatic organisms under environmental stress [42]. In this study, TEM was used to assess the impact of PVP-I exposure on mitochondrial integrity in the gill tissues of *M. rosenbergii*. As the primary site of oxidative phosphorylation and energy production, mitochondria are particularly vulnerable to oxidative damage [43]. Our results revealed clear concentration-dependent alterations in mitochondrial morphology, with recovery capacity diminishing as the toxicant concentration increased. At low exposure levels (1.14 mg/L), mitochondrial structure remained intact, and a slight increase in mitochondrial number was observed (Figure 6c). This likely reflects an adaptive response involving enhanced mitochondrial biogenesis to meet elevated energy demands and maintain redox homeostasis under sublethal stress [44]. Such remodeling may help minimize ROS leakage and preserve cellular function. In contrast, 4-day exposure to a high concentration (2.84 mg/L) caused pronounced mitochondrial damage, including reduced organelle density, vacuolization, cristae fragmentation, and matrix depletion, with the latter two likely directly suppressing energy metabolism efficiency and exacerbating cellular dysfunction [45] (Figure 6g). These structural alterations were accompanied by marked upregulation of apoptosis-related genes (*Bok*, *Caspase-3*, and *Cyt-c*), supporting the conclusion that mitochondrial dysfunction is a key driver of cell death under high-concentration PVP-I stress. This observation aligns with previous reports of mitochondrial ultrastructural damage in *Eriocheir sinensis* hepatopancreatic cells following cadmium exposure [46], suggesting that high concentrations of chemical stressors elicit conserved mitochondrial pathologies across diverse aquatic species. After a 7-day depuration period, partial restoration of mitochondrial architecture and density was observed in the low- (1.14 mg/L) and medium-concentration (1.89 mg/L) groups (Figure 6d,f), characterized by improved structural integrity and reduced vacuolization. However, the high-concentration group (2.84 mg/L) continued to exhibit significant mitochondrial lesions, including persistent vacuoles and disrupted cristae, indicating limited recovery and partial irreversibility of damage induced by elevated PVP-I exposure. These findings confirm that mitochondrial injury resulting from low-level PVP-I exposure can be at least partially reversed through depuration. In contrast, high-concentration exposure may induce irreversible cellular damage.

## 5. Conclusions

This study evaluated the effects of different concentrations of PVP-I exposure on immunity, antioxidant capacity, and apoptosis in *M*. *rosenbergii*. Low-concentration exposure caused minimal adverse effects, whereas high-concentration exposure induced oxidative stress and compromised the immune system. Additionally, high-concentration exposure triggered apoptosis in gill tissues, leading to mitochondrial damage and disintegration. After a 7-day recovery period in clean water, most damage was reversible; however, certain apoptotic injuries proved irreversible. Consequently, excessive use of PVP-I may be detrimental to *M. rosenbergii*, which emphasizes the importance of adhering to appropriate dosing protocols in aquaculture.

## Figures and Tables

**Table 1:** Primers used for qPCR in this study.

Primer Name	Sequence (5′→3′)	Sources
*β-actin*-F	CAGGGAAAAGATGACCCAGA	AY626840
*β-actin*-R	GGAAGTGCATACCCCTCGTA	
*ACP*-F	GCTTGGCTGTGACACTGATAAC	XM067128045.1
*ACP*-R	TCACAACTGACGAAGGTGTTTC	
*Hsp70*-F	TGACAAGGGTCGCCTCAGTA	[24]
*Hsp70*-R	CATTATCTTGTTGCGATCCTC	
*Toll*-F	TTCGTGACTTGTCGGCTCTC	KX610955.1
*Toll*-R	GCAGTTGTTGAAGGCATCGG	
*SOD*-F	GTGGCCTGGGACAATCGTTT	DQ121374.1
*SOD*-R	GTCTTATTTCGGCATCAGGC	
*CAT*-F	ACTTCATTACCCTGAGACCCG	HQ668089.1
*CAT*-R	TTTCCCTCAGCATTGACCAG	
*GSH-Px*-F	AGGGAAGGTGATTCTTGTGGA	FJ670566.1
*GSH-Px*-R	TTACAGGGGAAAGCCAGGA	
*Bok*-F	CGCCACAGTAGGAGAGAAGG	HG530759.1
*Bok*-R	TGAAAACGGCAATGGACATA	
*Caspase3*-F	TGAGGCACTGGTCTTGTCCAGAAT	HQ668093.1
*Caspase3*-R	GGCACTTGCATTGACTGCTGGATT	
*Cyt-c*-F	TGGGTGACGTAGAAAAGGGC	KU745282.1
*Cyt-c*-R	TGCCTTGTTAGCGTCAGTGT	

**Table 2:** Toxicity test results of PVP-I on *M. rosenbergii*.

Concentration (mg/L)	Average Mortality (%)				LC_50_ and 95% Confidence Interval (mg/L)				SC (mg/L)
	24 h	48 h	72 h	96 h	24 h	48 h	72 h	96 h	
0	0	0	0	0	8.49 (11.63~7.60)	6.90 (7.40~6.62)	6.08 (6.30~5.87)	5.67 (6.04~5.11)	1.37
5	3.33 ± 1.67 ^bc^	13.33 ± 1.67 ^c^	23.33 ± 1.67 ^d^	31.67 ± 1.67 ^d^					
5.5	8.33 ± 1.67 ^b^	16.67 ± 1.67 ^c^	31.67 ± 1.67 ^d^	40.00 ± 2.89 ^d^					
6	13.33 ± 1.67 ^ab^	23.33 ± 1.67 ^c^	48.33 ± 1.67 ^c^	58.33 ± 1.67 ^c^					
6.5	18.33 ± 1.67 ^a^	35.00 ± 2.89 ^b^	60.00 ± 2.89 ^b^	71.67 ± 1.67 ^b^					
7	21.67 ± 1.67 ^a^	58.33 ± 1.67 ^a^	76.67 ± 1.67 ^a^	100.00 ± 0.00 ^a^					

Note: Means with different superscripts in the same column indicate significant differences (*p* < 0.01).

## Data Availability

The data presented in this study are available on request from the corresponding author.

## Supplementary Materials

The following supporting information can be downloaded at: https://www.mdpi.com/article/10.3390/ani15152196/s1, Table S1: Disinfectants commonly used in prawn aquaculture; Table S2: Disinfectants with dangerous feedback for humans.

## Abbreviations

PVP-I	Povidone-iodine
LC_50_	Median lethal concentrations
SC	Safe concentration
Toll	Toll-like receptor
ACP	Acid phosphatase
*C. carpio*	*Cyprinus carpio*
TEM	Transmission electron microscopy
qPCR	Quantitative PCR
SOD	Superoxide dismutase
CAT	Catalase
GSH-Px	Glutathione peroxidase
TUNEL	Terminal deoxynucleotidyl transferase-mediated dUTP-biotin nick end labeling
Bok	B cell lymphoma 2 ovarian killer
*Cyt-c*	Cytochrome c
HSP70	Heat shock protein 70 gene
