# Supplementary material for: Toxic Effects of Povidone-Iodine on Macrobrachium rosenbergii: Concentration-Dependent Responses in Oxidative Stress, Immunosuppression, and Recovery Potential
Source: Animals (Basel). 2025 Jul 25;15(15):2196. doi: 10.3390/ani15152196 (PMC12345538; doi:10.3390/ani15152196)
Supplement: Supplementary file 1 [file animals-15-02196-s001.zip › animals-3734157-supplementary.pptx]

## Slide 1
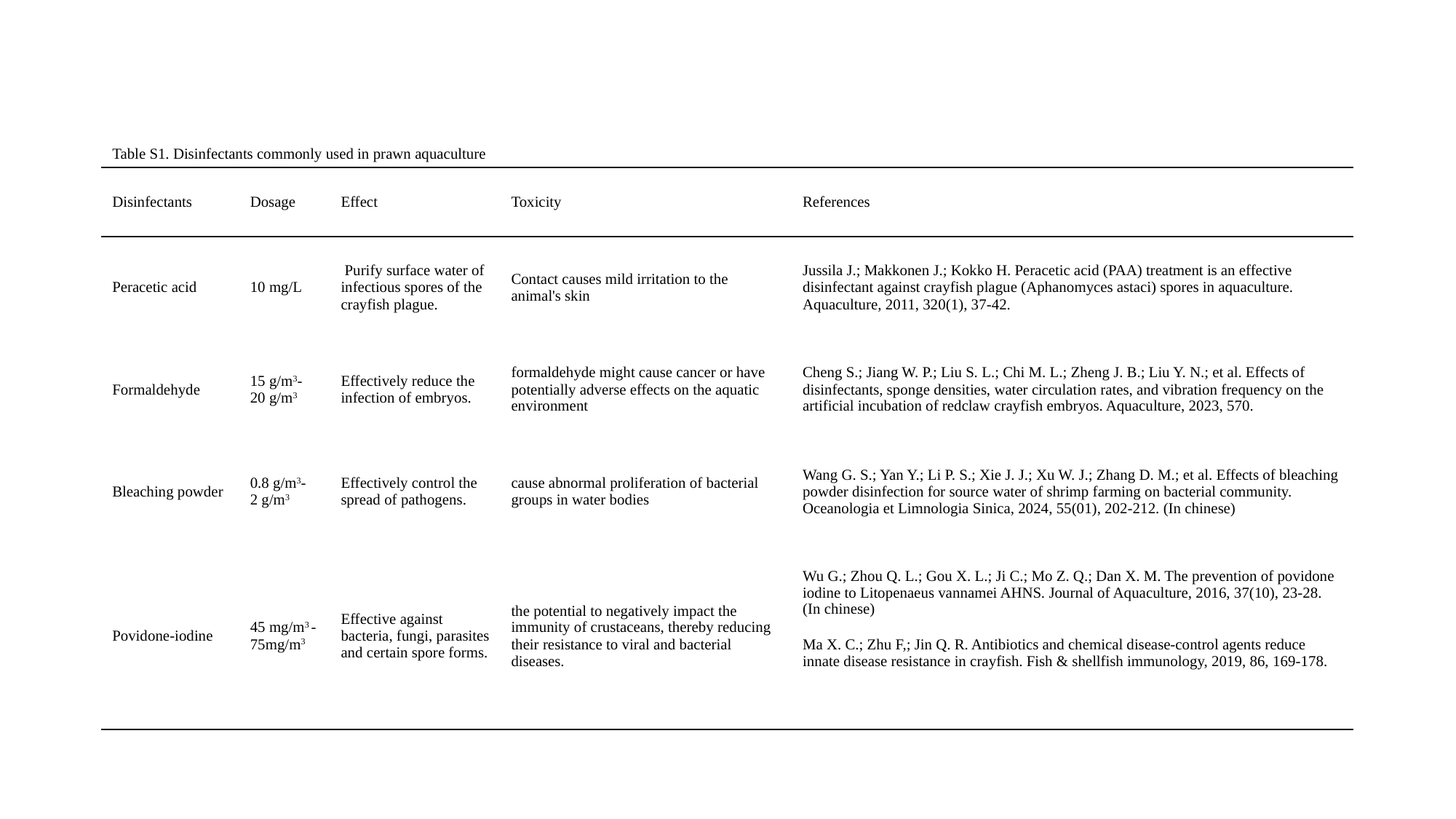

| Table S1. Disinfectants commonly used in prawn aquaculture | | | | |
| --- | --- | --- | --- | --- |
| Disinfectants | Dosage | Effect | Toxicity | References |
| Peracetic acid | 10 mg/L | Purify surface water of infectious spores of the crayfish plague. | Contact causes mild irritation to the animal's skin | Jussila J.; Makkonen J.; Kokko H. Peracetic acid (PAA) treatment is an effective disinfectant against crayfish plague (Aphanomyces astaci) spores in aquaculture. Aquaculture, 2011, 320(1), 37-42. |
| Formaldehyde | 15 g/m3- 20 g/m3 | Effectively reduce the infection of embryos. | formaldehyde might cause cancer or have potentially adverse effects on the aquatic environment | Cheng S.; Jiang W. P.; Liu S. L.; Chi M. L.; Zheng J. B.; Liu Y. N.; et al. Effects of disinfectants, sponge densities, water circulation rates, and vibration frequency on the artificial incubation of redclaw crayfish embryos. Aquaculture, 2023, 570. |
| Bleaching powder | 0.8 g/m3- 2 g/m3 | Effectively control the spread of pathogens. | cause abnormal proliferation of bacterial groups in water bodies | Wang G. S.; Yan Y.; Li P. S.; Xie J. J.; Xu W. J.; Zhang D. M.; et al. Effects of bleaching powder disinfection for source water of shrimp farming on bacterial community. Oceanologia et Limnologia Sinica, 2024, 55(01), 202-212. (In chinese) |
| Povidone-iodine | 45 mg/m3 - 75mg/m3 | Effective against bacteria, fungi, parasites and certain spore forms. | the potential to negatively impact the immunity of crustaceans, thereby reducing their resistance to viral and bacterial diseases. | Wu G.; Zhou Q. L.; Gou X. L.; Ji C.; Mo Z. Q.; Dan X. M. The prevention of povidone iodine to Litopenaeus vannamei AHNS. Journal of Aquaculture, 2016, 37(10), 23-28. (In chinese) Ma X. C.; Zhu F,; Jin Q. R. Antibiotics and chemical disease-control agents reduce innate disease resistance in crayfish. Fish & shellfish immunology, 2019, 86, 169-178. |

## Slide 2
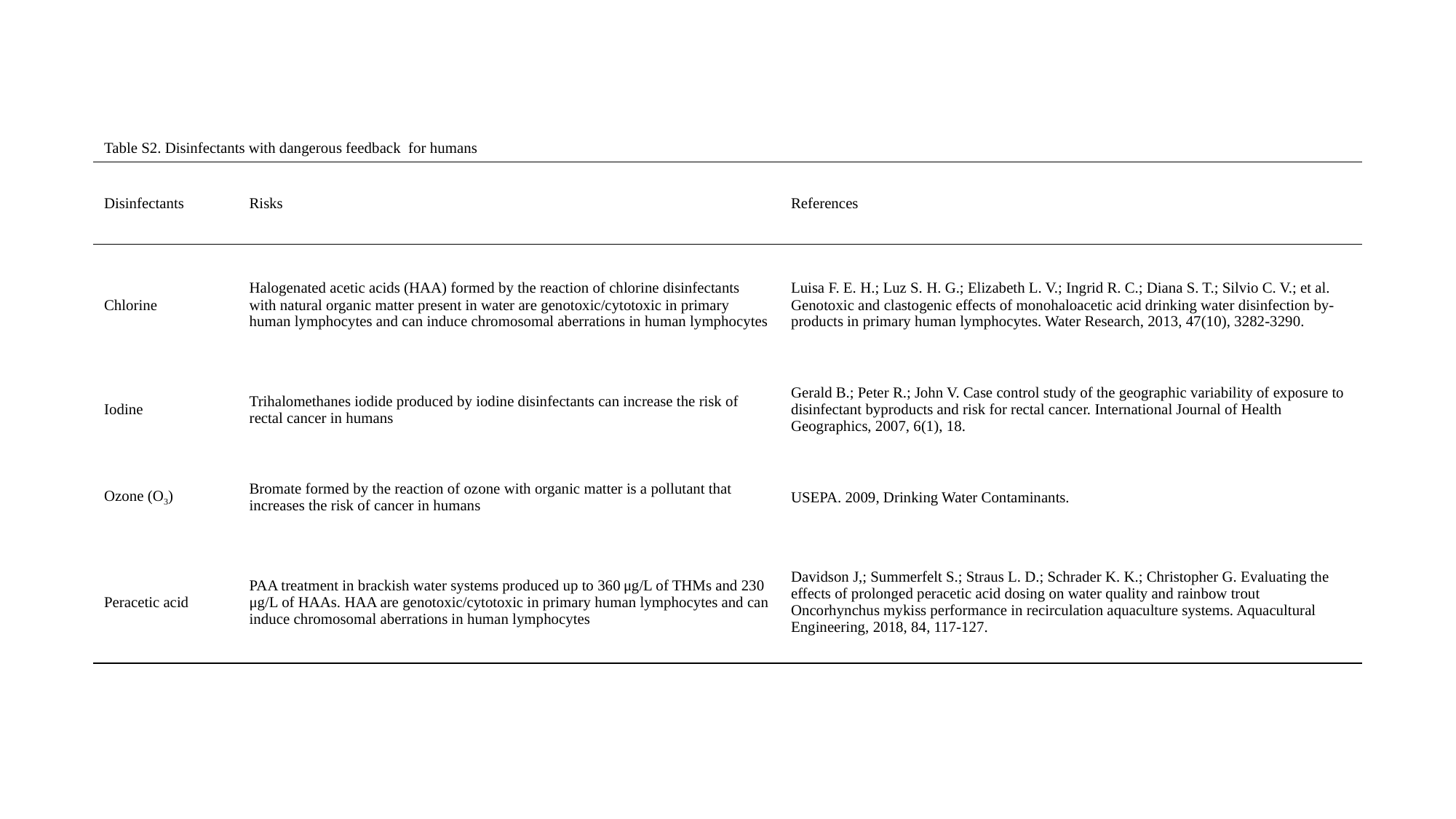

| Table S2. Disinfectants with dangerous feedback for humans | | |
| --- | --- | --- |
| Disinfectants | Risks | References |
| Chlorine | Halogenated acetic acids (HAA) formed by the reaction of chlorine disinfectants with natural organic matter present in water are genotoxic/cytotoxic in primary human lymphocytes and can induce chromosomal aberrations in human lymphocytes | Luisa F. E. H.; Luz S. H. G.; Elizabeth L. V.; Ingrid R. C.; Diana S. T.; Silvio C. V.; et al. Genotoxic and clastogenic effects of monohaloacetic acid drinking water disinfection by-products in primary human lymphocytes. Water Research, 2013, 47(10), 3282-3290. |
| Iodine | Trihalomethanes iodide produced by iodine disinfectants can increase the risk of rectal cancer in humans | Gerald B.; Peter R.; John V. Case control study of the geographic variability of exposure to disinfectant byproducts and risk for rectal cancer. International Journal of Health Geographics, 2007, 6(1), 18. |
| Ozone (O3) | Bromate formed by the reaction of ozone with organic matter is a pollutant that increases the risk of cancer in humans | USEPA. 2009, Drinking Water Contaminants. |
| Peracetic acid | PAA treatment in brackish water systems produced up to 360 μg/L of THMs and 230 μg/L of HAAs. HAA are genotoxic/cytotoxic in primary human lymphocytes and can induce chromosomal aberrations in human lymphocytes | Davidson J,; Summerfelt S.; Straus L. D.; Schrader K. K.; Christopher G. Evaluating the effects of prolonged peracetic acid dosing on water quality and rainbow trout Oncorhynchus mykiss performance in recirculation aquaculture systems. Aquacultural Engineering, 2018, 84, 117-127. |
